# Pulmonary Langerhans Cell Histiocytosis Associated with Bronchogenic Carcinoma

**DOI:** 10.7759/cureus.6634

**Published:** 2020-01-12

**Authors:** Muhammad F Khaliq, Muhammad M Noorani, Syed Maaz Tariq, Ashish Koirala, Hesham Mohamed

**Affiliations:** 1 Internal Medicine, Charleston Area Medical Center, West Virginia University-Charleston Division, Charleston, USA; 2 Hospital Medicine, Baptist Medical Center South, Montgomery, USA; 3 Internal Medicine, Jinnah Sindh Medical University, Karachi, PAK; 4 Internal Medicine: Pulmonology/Critical Care, West Virginia University School of Medicine, Morgantown, USA; 5 Internal Medicine: Pulmonology/Critical Care, Charleston Area Medical Center, Charleston, USA

**Keywords:** pulmonary langerhans cell histiocytosis, lung adenocarcinoma, mycobacterium avium complex

## Abstract

Pulmonary Langerhans cell histiocytosis (PLCH, pulmonary eosinophilic granuloma) is a rare disease of clonal dendritic cells that primarily affects adults who smoke cigarettes. PLCH association with other malignancies is rarely reported. Herein, an unusual case of PLCH is presented with synchronous lung adenocarcinoma. A 76-year-old woman and chronic smoker was admitted for persistent dyspnea and productive cough, and had a left lower lung mass detected by computed tomography. She underwent bronchoscopy with biopsies. Histopathological analysis was negative, but cultures grew Mycobacterium avium complex. She subsequently underwent lobectomy and was found to have papillary adenocarcinoma with PLCH in the surrounding lung nodules.

## Introduction

Pulmonary Langerhans cell histiocytosis (PLCH, pulmonary eosinophilic granuloma) is an orphan disease of clonal dendritic cells that primarily affects young smokers. Several cases reported in the literature highlight coexistence of PLCH and other malignancies, although no causal relationship has been established [1,2]. There are no etiological factors yet identified in the literature which could be the root cause of the problem. This is a difficult to diagnose condition and almost always presents as an incidental finding. Coexistence of lung cancer can complicate the course the disease. Low prevalence combined with no clear etiology and no sufficient data poses a challenge to the clinician on what treatment option to choose. Here we present a case of PLCH that presented with synchronous adenocarcinoma of the lung. 

## Case presentation

A 76-year-old woman presented with a one-year history of gradually progressive chronic shortness of breath and productive cough associated with significant weight loss. She had a 120 pack-year smoking history and was dependent on oxygen therapy for chronic obstructive pulmonary disease. She had a past medical history of stage III chronic kidney disease, coronary artery disease with stenting, gastroesophageal reflux disease, and hypertension. 

**Figure 1:**
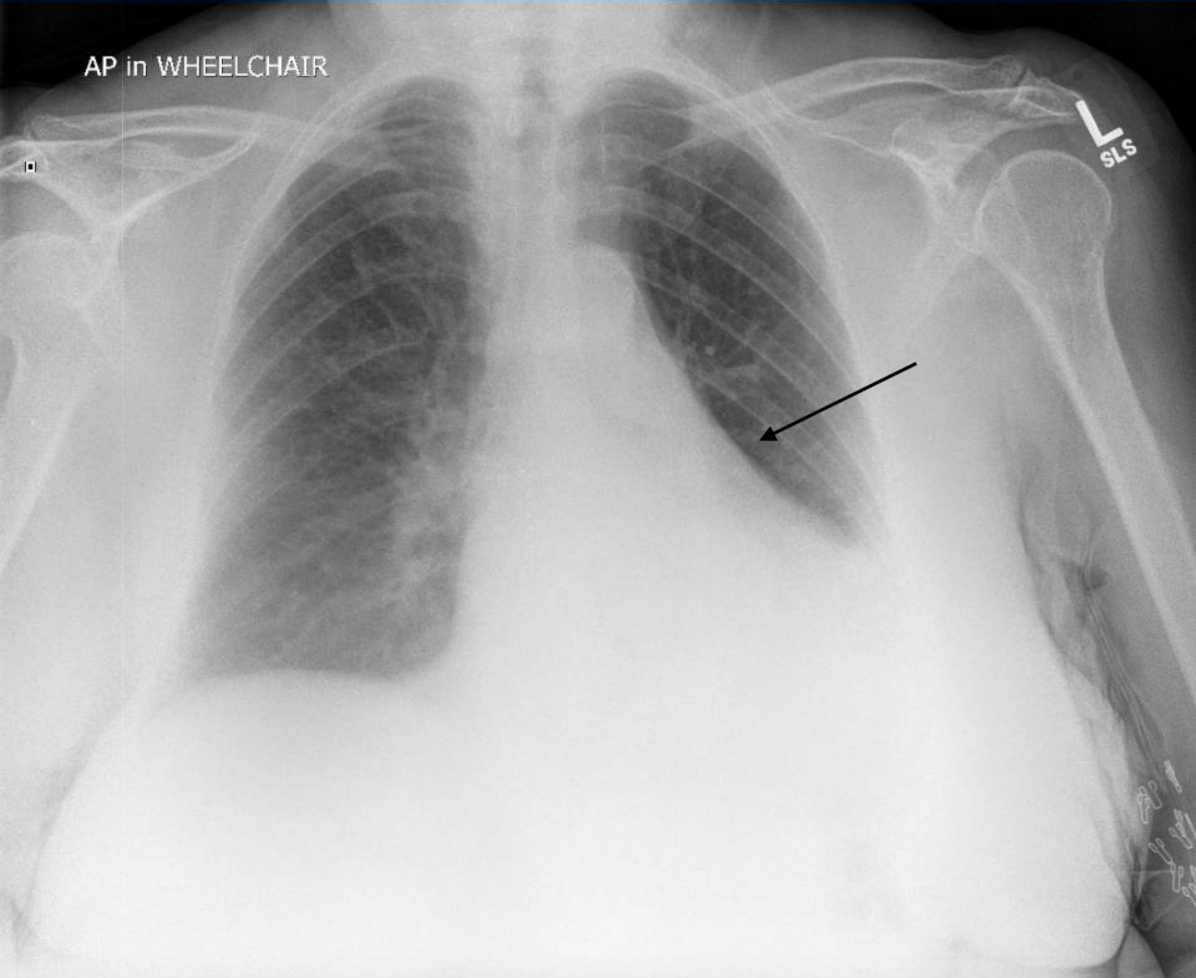
Left basilar consolidation seen on chest X-ray.

**Figure 2:**
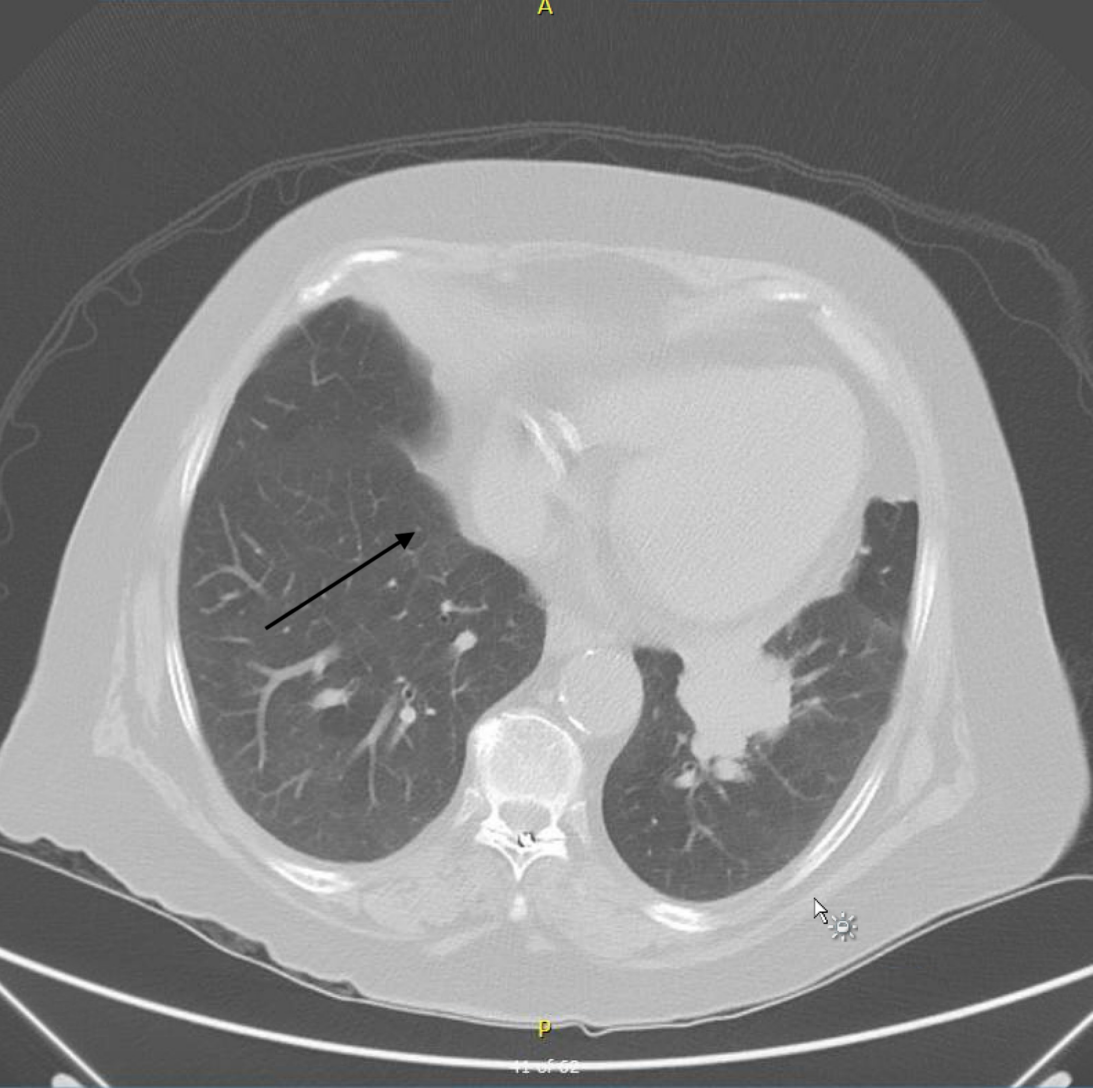
Chest CT showing mass-like consolidation surrounded by nodules.

Her chest radiograph showed left basilar consolidation. She was initially treated with antibiotics, bronchodilators, and inhaled corticosteroids. A computed tomography (CT) scan revealed an irregularly shaped mass in the inferior left lower lobe measuring 4.4 x 4.5 cm and suspicious for malignancy (Figures 1, 2).

There was no lymphadenopathy noted on the CT scan. Bronchoscopy with endobronchial ultrasound was performed that did not reveal malignancy. However, bronchoalveolar lavage fluid (BALF) cultures grew Mycobacterium avium complex (MAC). 

**Figure 3:**
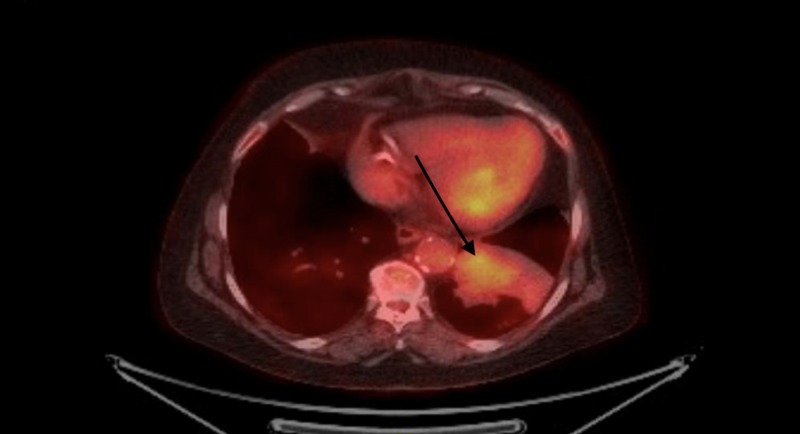
Positron emission tomography examination revealing worsening consolidation within the left lower lobe with moderate intensity standardized uptake value maximum of 4.3 noted within the infrahilar region. No abnormal uptake noted in hila, mediastinum, or abdomen.

A positron emission tomography (PET)-CT scan to further characterize the underlying pathology showed increased uptake in the left lower lobe bronchus suspicious for malignancy (Figure 3).

A surgical lung biopsy and a lobectomy were performed. Histopathological examination of the specimen revealed papillary adenocarcinoma. Biopsied nodules contained numerous CD1a-positive histiocytes demonstrating a Langerhans cell phenotype that, together with the morphological features, was diagnostic for PLCH. Lymph node biopsy was negative for malignancy.

Echocardiography to monitor pulmonary pressures revealed good systolic function with an ejection fraction of 55%-60% and right ventricular systolic pressure of 34 mmHg. Due to her multiple comorbidities, along with personal and family preferences, adjuvant chemotherapy was not administered. Surveillance CT was advised. 

## Discussion

PLCH is a rare interstitial disease that usually affects young to middle-aged adult smokers with no gender predilection [3]. PLCH can manifest with a normal to mild obstructive pattern on pulmonary function tests and diffusion impairments [3,4]. Histopathologically, PLCH is characterized by granulomatous infiltration of the distal bronchial walls by proliferating Langerhans cells and an eosinophilic infiltrate. Inflammatory changes initially manifest as nodules and later as thick and thin walled cysts, with fibrosis seen in advanced cases on chest radiography [5]. Cyst burden correlates with lung function abnormalities and predicts functional decline [6].

No etiological factors have been identified, but retrospective analyses have reported that >90% of cases occur in either current or previous smokers [7]. Familial PLCH is an extremely rare condition, with only few cases reported in the literature [7,8]. It is often difficult to establish with certainty the age of onset, since a quarter of patients are asymptomatic and are only diagnosed incidentally on chest imaging for other purposes [1,3,9]. This can result in an overlooked diagnosis for many years. If symptomatic, patients can present with non-productive cough and dyspnea [6]. Occasionally, constitutional symptoms are present such as weight loss, fever, night sweats, and appetite loss. Another common presentation is sudden-onset dyspnea due to pneumothorax. Hemoptysis is uncommon and, if present, carcinoma should be ruled out [6]. 

Chest radiographs can be normal but there are typically reticulonodular opacities and interstitial abnormalities in the upper and middle lobes. CT demonstrates thin- and thick-walled cysts, which can later cause fibrosis. This nodularity is often difficult to differentiate from metastasis, thus necessitating lung biopsy. 

Previously, 5% CD1a-positive cells (a histiocyte marker) in BALF were considered diagnostic of PLCH, but increased CD1a-positive cells are also seen in asymptomatic smokers and can represent activated M2 macrophages, so the sensitivity and specificity of this test are poor [10,11]. Lung biopsy can help in establishing a definitive diagnosis but has its own complications such as pneumothorax, bleeding, and respiratory failure. In asymptomatic smokers with highly characteristic lesions, a presumptive diagnosis can be made. Biopsy can be deferred and lesions can be followed closely with serial monitoring. Recurrent pneumonia in our patient with mass on CT scan and MAC on BALF prompted surgical lung biopsy. The need to establish a definitive diagnosis should be balanced with the risks associated with surgical biopsy. In cases of extrathoracic involvement, lesions in the bone or skin may confirm the diagnosis if pulmonary manifestations are consistent with PLCH [1,2].

Several case reports have highlighted an association between PLCH and other malignancies including lung cancers [12-14]. Predisposing risk factors include prolonged exposure to cigarette smoking, genetic abnormalities, or prior chemotherapy [1]. A considerable number of patients with PLCH develop adenocarcinoma on longitudinal follow-up. It is difficult to say whether PLCH increases the risk of malignancy or whether smoke exposure results in the development of two synchronous but distinct pathologies. In our case, no fibrosis was observed and the adenocarcinoma was more central and scar carcinomas are known to present peripherally [15]. Regardless, we think it is prudent to counsel patients regarding smoking cessation, since it is a reversible risk factor with resolution of radiographic findings within one year [16,17].

PET-CT is effective for staging and evaluating responses to therapy in PLCH. Those who are diagnosed with PLCH should have close clinical and radiographic follow-up since they are usually heavy smokers with a high risk of lung cancer development. Patients should also be counseled about this association. 

Due to the very low prevalence and variable course of the disease, no reliable data currently exist on effective treatment modalities. Some patients develop progressive disease, and those who complain of unexplained dyspnea with decreased diffusion capacity should be screened for pulmonary hypertension by Doppler echocardiography with confirmation by right heart catheterization as it has shown to be a predictor of prognosis with an impact on survival [18]. Lung transplantation is a therapeutic option for patients with advanced PLCH [19].

## Conclusions

PLCH is associated with an increased risk of bronchogenic carcinoma and lymphoma. A high index of suspicion is required to diagnose synchronous disease. There is no standard treatment for PLCH. Smoking cessation is essential to reverse PLCH. As PLCH and malignant tumors are associated, patients with PLCH may benefit from screening for associated malignancies
